# Nanoplastics as Gene and Epigenetic Modulators of Endocrine Functions: A Perspective

**DOI:** 10.3390/ijms26052071

**Published:** 2025-02-27

**Authors:** Massimo Aloisi, Anna Maria Giuseppina Poma

**Affiliations:** Department of Life, Health and Environmental Sciences, University of L’Aquila, I-67100 L’Aquila, Italy; massimo.aloisi@univaq.it

**Keywords:** nanoplastics, gene modulators, endocrine functions, breast milk, breast cells, placenta

## Abstract

Nanoplastics (NPs) represent a major challenge in environmental contamination resulting from the physical, chemical, and biological degradation of plastics. Their characterization requires advanced and expensive methods, which limit routine analyses. The biological effects of NPs depend on their chemical and physical properties, which influence toxicity and interactions with biological systems. Studies in animal models, such as *Daphnia magna* and *Danio rerio*, show that NPs induce oxidative stress, inflammation, DNA damage, and metabolic alterations, often related to charge and particle size. NPs affect endocrine functions by acting as endocrine disruptors, interfering with thyroid and sex hormones and showing potential transgenerational effects through epigenetic modifications, including DNA hyper- and hypomethylation. Behavioral and neurofunctional alterations have been observed in *Danio rerio* and mouse models, suggesting a link between NP exposure and neurotransmitters such as dopamine and serotonin. Despite limited human studies, the presence of NPs in breast milk and placenta underscores the need for further investigation of health effects. Research focusing on genetic and epigenetic markers is encouraged to elucidate the molecular mechanisms and potential risks associated with chronic exposure.

## 1. Introduction

**Figure 1:**
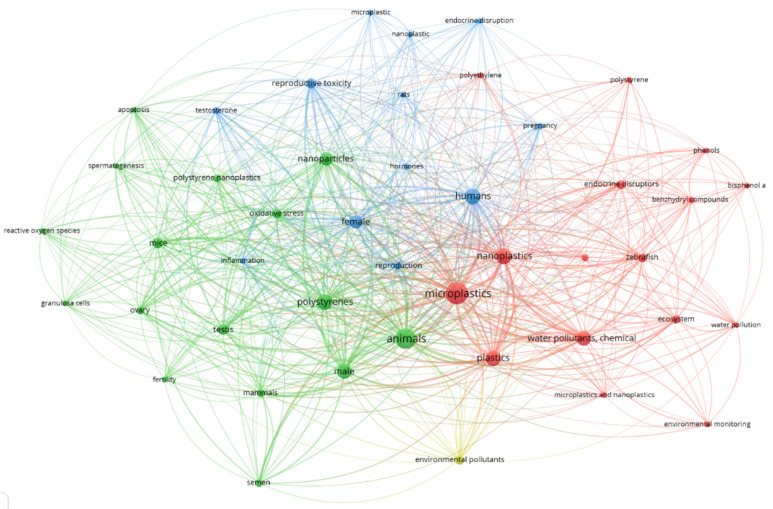
Bibliometric association between nanoplastics and endocrine functions. The figure shows the strength of links between the most relevant keywords looking for “nanoplastics” AND “endocrine” on PubMed. A total of 104 results were found against more than three thousand results from typing only “nanoplastics”, indicating that it is still an overlooked topic. In the green cluster, NPs are related specifically to oxidative stress and inflammation in mice and mammals. From reading the keywords, it can be seen that the main outcomes are linked to fertility, sperm and oocytes functionality, and ovary and testis integrity. The blue cluster focuses on generic “hormones” and on “testosterone”. These two words are also connected with “humans”, “endocrine disruptors”, and “pregnancy”, indicating that reproduction is still the main topic when NPs are associated with humans. If the environmental effects of NPs are considered, the red cluster shows that *zebrafish* is the principal animal model and that plastic leachates are the prevalent molecules in these types of studies. The yellow cluster composed of only one keyword, “environmental pollutants”, is connected to the green and red ones, confirming that the relation between environment and human hazards is a topic that remains poorly investigated.

Plastic residues dispersed in the environment represent one of the biggest pollution-related issues of contemporary age. Environmental concentrations are increasing, and they have been found from urban to extreme areas such as glaciers and oceanic floor [1,2,3]. Plastic residues undergo physical, chemical, and biological degradation, forming smaller fragments called macroplastics if bigger than 5 mm, microplastics (MPs) if the size is comprised between 100 nm and 5 mm, and nanoplastics (NPs) if smaller than 100 nm [4,5,6,7,8]. Determining their amount in the environment can be challenging in the case of NPs. While MPs can be sampled using nets with an appropriate mesh diameter, NPs are too small to be trapped [9,10]. Advanced techniques like mass spectrometry or scanning electron microscopy (SEM) are required to characterize NPs [11]. Of course, considering the required skills and the expensiveness of these methods, they are not suitable for routinary analysis and screening. These limits also influence the choice of sizes and concentrations used in experiments that are consequently based on the few papers determining these parameters [12]. Chemical characterization is the starting point when studying nanomaterials and consequently NPs. Considering the technological limitations, it appears too complex to elaborate appropriate measures to mitigate the problem. Proper regulation is the most efficient way to achieve this goal, but it remains challenging due to the lack of scientific information. The heterogeneity of plastic polymers makes it difficult to summarize the toxic effects. Moreover, public perception is variable depending on cultural variables all over the globe. A unified global agreement is the ideal solution to synchronize worldwide legislation and align the negative outcomes connected to this topic. Additional research on innovative technologies to sample nanoplastics may help [13]. In addition to NPs alone, they can present chemicals bound to their surface or positive/negative charge influencing their kinetic properties and interactions with biological systems [14]. The study of the synergistic effect of compound-bond NPs is crucial to understanding the biological effects that all the pollutants to which humans are simultaneously exposed can cause. In fact, the expression “Trojan horse effect” precisely indicates the fact that some compounds take advantage of the higher ability that nanomaterials have in being absorbed by a biological organism [15,16]. In particular, hydrophobic organic compounds (polychlorinated biphenyls, polycyclic aromatic hydrocarbons, and other benzene ring derivatives), hydrophilic organic compounds (perfluorooctanesulfonate and perfluorooctanesulfonamide), and metals were found adsorbed bymicro- and nanoplastics [17]. In all of these papers, exposure has always been assessed with NPs and other compounds together, and to our knowledge, there are few works that have investigated the role of NPs alone. Indeed, the purpose of this perspective is to bring attention to the need to determine their effects alone. The endocrine tissues previously discussed are mainly the thyroid, the reproductive tissues, and behavior-related hormones, still considering NPs and other pollutants [18,19]. Few studies are focused on the direct effects of NPs on biological targets such as receptors and the consequent endocrine function. The major portion describes the effects of plastic leachates but not the role of the particles alone (Figure 1). From this perspective, we included some incisive examples of how NPs can induce toxic effects on major model organisms. Articles were selected from PubMed through December 2024 using the keywords “nanoplastics” AND “referred model organism” (to be replaced with models of interest). For length limitations, only a few representative articles were included, despite there being many more present in scientific literature. We prioritized the most recent papers, and we apologize to the colleagues whose work was not included in this paper.

### Biological Effects

**Figure 2:**
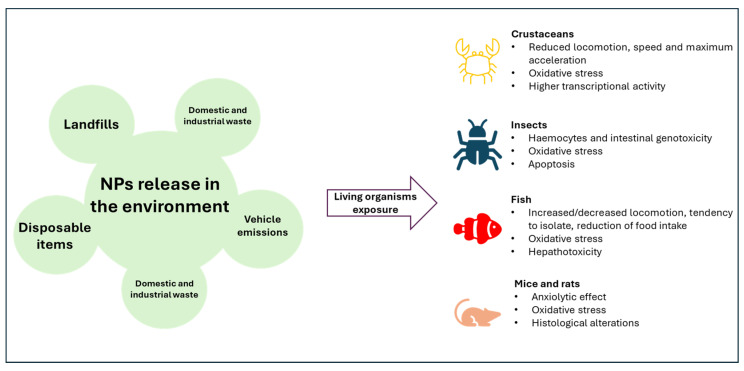
Biological effects of nanoplastics (NPs). The figure shows the main biological effects induced by NPs in different animal models used to infer effects on humans. Oxidative stress and inflammation are the main ones found to be conserved throughout the different species.

Determining the impact of NPs on living organisms is fundamental not only for unveiling the environmental consequences of plastic pollution but also to infer translationally the hazards to humans. Therefore, studies on animal models are the main path to clarify human risk. Filter-feeding animals are considered environmental sentinels because of their habit of feeding on suspended molecules. Therefore, they tend to accumulate in their bodies whatever is found dispersed in the environmental matrices they live in [20]. In this case, a suitable model is represented by *Daphnia magna* thanks to interesting features such as a short life cycle, cheap maintenance in a laboratory, and a transparent body that allows anatomical considerations [21]. Neutral 100 nm polystyrene NPs (PSNPs), positive-charged PSNPs (PS-NH2), and negative-charged PSNPs (PS-COOH) were proven to induce different effects on *D. magna.* The LC50 (lethal concentration for 50% of the organisms tested) was 5.24 mg/L for neutral ones, 8.56 mg/L for PS-NH2, and 20.2 mg/L for PS-COOH. Behavioral tests showed that PS-NH2 was the most effective in reducing locomotor performance, such as distance traveled, speed, maximum acceleration, and percentage of movement. Nevertheless, neutral PSNPs induced the highest oxidative stress and increased GSH (glutathione) expression. Neutral PSNPs also induced higher phosphorylation of p38 (p-p38) and c-Jun N-terminal kinase (p-JNK), confirming higher transcriptional activity. After 21 days of exposure, 50 nm PSNPs (0.5 μg/mL) also increased lipid and caloric content [22,23]. These results confirmed the relevance of NP properties in determining biological effects on animals. Positively charged NPs proved to be more toxic due to the interactions with the negatively charged cell membranes, with consequent higher internalization rates. This difference was also confirmed on other organisms, such as the sea urchin *Paracentrotus lividus*, in which 50 µg/mL of PS-NH2 induced severe embryotoxicity (LC50 3.85 μg/mL 24 hpf and 2.61 μg/mL 48 hpf) that led to embryo lethality and severe structural anomalies. On the other hand, PS-COOH in the same conditions did not show any effect [24]. Studies on the genetic model of *Drosophila melanogaster* proved that different sizes and polymers can induce different grades of severity, highlighting the relevance of NP chemical composition. On larvae, PSNPs of 50 and 100 nm induced oxidative stress and DNA damage detected with the comet assay on hemocytes and tissue damage and genotoxicity specifically on the intestine. NPs with larger diameters, such as 200 and 500 nm, still induced the same effects, but the intensity was reduced by at least 10% [25,26]. Polyethylene terephthalate NPs (PETNPs) instead reduced the expression of stress-related genes such as *hsp70*, reduced oxidative stress, and had less DNA damage when compared to PSNP exposure [27]. Apparently, different polymers induced different levels of cellular stress in *D. melanogaster*. Organ-specific damage to the liver was proven on *zebrafish* (*Danio rerio*). PSNPs (70 nm, 20 mg/L) induced liver inflammation and increased lipid accumulation in this tissue [28]. The same NP size but reduced concentrations (0.5, 1.5 mg/L) caused the same effects [29]. On rodents, it has been demonstrated that NPs cause general oxidative stress and inflammation [30]. Fan et al. [31] gave wild-type mice 86 nm PSNPs orally at doses of 0 mg/kg, 5 mg/kg, and 15 mg/kg for 20 weeks. After one week, they found that body weight had decreased, while plasma glucose levels had increased. There were no variations in insulin levels. Reduced adipocyte area was observed, which was consistent with lower lipid storage in the other parameters. In another study, the exposure of mice to PSNPs (5 mg/kg/d PS-NPs) caused hepatic oxidative stress by suppressing the nuclear factor erythroid-derived 2-like 2 (NRF2) antioxidant pathway and its downstream antioxidase expression, as well as by inducing an excessive amount of reactive oxygen species. Furthermore, PSNPs increased the expression of caspase-1, NLRP3, and IL-1β in addition to activating NF-κB, indicating that PSNPs caused hepatocellular inflammatory damage [32]. Overall, these results show that NP exposure induces toxic effects with different grades of severity depending especially on the NPs’ charge and size. Inflammation and oxidative stress are the main first cellular responses that usually lead to DNA damage through oxidation [33], cytotoxicity [34], and nutritional and metabolic alterations (Figure 2) [35]. Even more challenging than clarifying specific outcomes deriving from NP exposure is elaborating a possible treatment to reduce the toxic effects. Some nanomaterials have been revealed to be useful in attenuating oxidative stress and inflammation. For instance, Zhao et al. [36] reported that fingolimod hydrochloride (FTY720), an FDA approved drug, is able to reduce inflammation following ischemic events by altering Cebpb-regulated NLRP3 inflammasome activation and CXCL2 chemokine production, hence converting microglia toward anti-inflammatory phenotypes. In addition, numerous compounds from natural sources or nutrients from diet have demonstrated antioxidant effects [16]. Nowadays, a diet rich in these molecules may be the best way to prevent adverse effects over a long-term period. Considering the reported toxicity of NPs on relevant endocrine tissues like liver and intestine observed in the main animal models, the effect of NPs on endocrine signaling assumes a central role. Few studies have investigated this phenomenon. This perspective will describe studies unveiling the connection between NPs and endocrine signaling, focusing on future possible applications to deconvolute the link between gene expression and epigenetic alterations of genes related to endocrine functions, if present.

## 2. Nanoplastics as Endocrine Disruptors

Endocrine disruptors (EDs) are defined by the FDA (U.S. Food and Drug Administration) as “an agent that interferes with the synthesis, secretion, transport, binding, or elimination of natural hormones in the body that are responsible for the maintenance of homeostasis, reproduction, development and/or behaviour” [37]. The property of influencing hormone signaling represents the mechanism by which EDs interact with biological systems, influencing organ functioning. Pesticides are among of the most studied and well-known EDs [38]. For instance, DDT (dichlorodiphenyltrichloroethane) was one of the most used pesticides in agriculture, public and private gardens, beaches, and public areas. It became the symbol of the war against mosquitoes during the 20th century until Dr. Rachel Carson published the book *Silent Spring* in 1962 reporting the toxic effects of pesticides, DDT included [39]. Today, it is known that DDT can affect the thyroid, estrogen, androgen, rennin-angiotensin, insulin, and neuroendocrine systems, all of which can have a direct impact on the body’s metabolic, cardiovascular, and reproductive systems [40]. The history of DDT highlights the necessity of assessing the potential endocrine disrupting effects of pollutants before their effects become irreversible. In this perspective, we are going to report and comment on the few results related toNPs and endocrine alterations focusing on gene modulations, when available. More studies have been conducted on plastic leachates that are employed to modify plastic products’ final properties, but little attention has been given to NPs alone. The main goal of this perspective is to revalue this aspect for future research. Particles with a diameter of 100 nm or less have already been proven to influence endocrine pathways. Table 1 shows the main effects induced by different nanoparticles on endocrine functions that may be helpful to direct future studies on nanoplastics chosen from among the most recent papers found on PubMed.

### 2.1. NPs and Reproduction

There are few recent studies assessing the effects of NPs on reproductive systems. Sex hormones of marine fish *Oryzias latipes* were proven to be altered by PSNP exposure (100 nm, 0, 10, 10^4^, and 10^6^ particles/L for 90 days) [54]. High NP exposure (10^6^ particles/L) impaired spermatogenesis and oogenesis, according to gonadal histology, suggesting that the gonad matured later than expected. Oxidative stress and immune response biomarkers were also evaluated. In testes, lysozyme and malondialdehyde resulted in being significantly less expressed than the controls at the highest concentration; glutathione peroxidase, superoxide dismutase, and catalase resulted in being less expressed at 10^4^ particles/L. At the highest concentration, only superoxide dismutase resulted in being overexpressed. In ovaries, all the enzymes were downregulated at all concentrations. Considering the reduced expression of antioxidant enzymes, it is unlikely that oxidative stress led to developmental delay. Or, on the contrary, high initial oxidative stress could have depleted the antioxidant reservoir in long-term exposure. Gene expression analysis all along the exposure time could unveil signaling modifications induced by PSNPs causing the reported reductions. In addition, the developmental delay observed histologically could be explained with sex hormone analysis. Their reduced expression could not only slow down spermatogenesis and oogenesis but also reduce transcriptional activity. In *Caenorhabditis elegans* exposed to 100 nm PSNPs (0, 1, 10, 100, and 1000 mg/kg dry weight, for 96 h), the same gonad atrophy was detected [55]. A significant reduction of intracellular ATP and egg production in ovaries was observed alongside an increase in the range of 14–31% compared to controls of *ced-3* mRNA. This is a biomarker of apoptosis considered representative of apoptotic induction [56]. The analysis of sex hormones, both genetic and biochemical, could explain, in case of reduction, these effects. Examples of genetic markers studied in *zebrafish* to determine the impact of NPs on female reproduction include *sod*, *gpx*, nrf2, inos, *ucp2*, and *atp6* (oxidative stress); *nfkβ*, *tnfα*, *il-10*, *ikβ*, *gdf9*, and *bmp15* (immune response); and *gadd45*, *rad51*, *p53*, and *bcl2* (DNA damage and apoptosis) [57]. The authors exposed *zebrafish* oocytes to PSNPs for 6 h (100 ng/mL and 400 ng/mL). All the markers resulted in being overexpressed, particularly with the 400 ng/mL concentration. The only exceptions were *atp6* and *ucp2*, which resulted in being more expressed in the 100 ng/mL condition. This difference highlights the need for gene expression to differentiate the pathway that various concentrations could alter to induce a toxic effect. In male rats, 50 nm PSNPs administered for 35 days (0, 1, 10 mg/Kg) induced disruption of acrosome biogenesis, leading to reduced sperm functionality [58]. Moreover, genetic markers of acrosome biogenesis were investigated: *Gba2*, *Pick1*, *Gopc*, *Hrb*, *Zpbp1*, *Spaca1*, and *Dpy19l2.* In particular, *Gopc* and *Dpy19l2*, which are employed in vesicles transport from the Golgi apparatus to acrosomes, were significantly downregulated. Some authors discussed these results, stating that the still unclear link between autophagy and the two markers could be the basis of the mechanism behind. We add to the comment that sex hormones could be a connecting point considering their proven role in regulating autophagy in gametes and their alterations induced by NPs in other model organisms [59]. Several studies reported multi- and transgenerational effects following NP exposure [60]. Despite this, there are only a few unraveled epigenetic signatures that could be useful to track hereditable marks. This topic acquires importance considering that NP residues have been found in human breastmilk and placenta, proving that as humans, we are exposed since infantry or even before being born [61,62]. Exposing copepods (F0) to 50 nm PSNPs (10 mg/L) caused reproductive impairments in the F1 and F2 generations, even though they were never exposed to NPs [63]. These effects were correlated with hypermethylation of specific genes in the generations that were not exposed to NPs. Specifically, heat shock protein 70 (*Hsp70*), superoxide dismutase (*CuZn SOD*), calmodulin 3 (*CALM3*), cell death-inducing DFFA-like effector c (*CIDEc*), and *p53* genes were commonly found hypermethylated in both multi- and transgenerational conditions. With a network analysis, the authors concluded that DNA methylation could be associated with oxidative stress and calcium homeostasis-related processes in disrupting reproduction. This paper is an exemplary study of the utility of epigenetic markers. Correlating phenotypes and epigenetic signatures allowed suggestions of what pathway was implicated in NPs’ toxic effects.

### 2.2. NPs and Human Breast Epithelial Cells

NPs can affect the homeostasis of breast cells. They are an important source of endocrine factors, and they are also a relevant target of hormones [64]. NPs have already been found in breastmilk, implying their presence in the tissue [65]. Park et al. [65] showed that MDA-MB-231 cells (human epithelial cell line) exposed to polypropylene (PP) for 24 h (in this case they were MPs of 16.4 µm of diameter) were characterized by altered expression of relevant genes. In particular, gene expression of *TMBIM6*, *AP2M1*, and *PTP4A2* (correlated with cancer progression and cell cycle) increased, while levels of *FTH1* (coding for the ferritin heavy chain) were decreased. The increased expression of genes implicated in metastasis clearly indicates that PP can alter cell cycle and motility, showing that genes related to these aspects may be considered. Schnee et al. [66] analyzed the effects of PSNPs on M13SV1_Syn1-DSP8-11 breast epithelial cells and HS578T-DSP1-7 and MDA-MB-231-DSP1-7 human breast cancer cells. PSNPs were still in the MPs range. Detected effects were moderate, with a slight induction of proliferation and cell migration in cancer cells, supporting the previous results. In addition, this work suggests that the presence of NPs may aggravate the phenotype of an existing disease supporting the necessity of testing how NPs interacts with human pathologies. The only study, to our knowledge, that systemically investigated if NPs interacts physically with the estrogen receptor is from Božičević et al. [67]. They tested PP, PS, and PE NPs (180 nm, 25 nm, and 350 nm) on the T47D-KBluc cell line (isolated from a human carcinoma of the mammary gland). These cells were transfected with luciferase as a reporter enzyme to emit light when the estrogen receptor is activated. They observed that PE was the most affecting polymer, inducing luminescence at all concentrations, with a peak at the highest (10, 1, 0.1, 0.01, 0.001 mg/L). PP induced luminescence only with 10 and 5 mg/L, while PS was the only one not causing a significant change, but there was a trend with a peak at the highest concentration. These results show the importance of physical interaction and that NPs are able to directly interact with receptors related to endocrine functions. Moreover, PE was the most active,, even if the size was the highest, showing that chemical composition may be more relevant, in this case, than size. More studies could be useful to clarify the relationship between these important factors.

### 2.3. NPs and Behavior

Surprisingly, NPs were proven to alter behavior and neural functions [68]. It is not easy to determine biomarkers for such a complex topic, considering that behavior can be regulated by a huge plethora of endocrine signals. Therefore, the study of how NPs can alter neural functions and behavior is complex, and it needs to take into consideration a plethora of pathways originating from different tissues. Sarasamma et al. [29] exposed zebrafish to 70 nm PSNPs (0.5 ppm, 1.5 ppm, and 5 ppm for 7 and 30 days and then 7 weeks). Alongside inflammation and oxidative stress in liver, altered energy metabolism was detected. In particular, a significant reduction in ATP was measured. The surprising results are related to the altered behavior of fish exposed to PSNPs. Low concentrations caused fish to dive into the tanks’ bottoms, while the highest concentrations induced hyperactivity and abnormal swimming. The first interesting result was the downregulation of the acetylcholinesterase (AchE) in the hippocampus of exposed organisms. The knockdown of the *Ache* gene in mice induces anxiety-like behaviors [69]. The reduction in AchE was accompanied by significant reductions in dopamine, melatonin, serotonin, vasopressin, and oxytocin levels. These results could explain the abnormal swimming activity consisting of increased locomotor activity and exploration behavior and decreased predator avoidance and aggressiveness. Indeed, oxytocin and vasopressin together are essential parts of the hypothalamo-neurohypophysial system regulating social behaviors and exploration of new environments [70]. Serotonin and dopamine are involved in the regulation of several developmental, behavioral, and physiologic processes, including anxiety and affective states, reinforcing the previous results [71]. In addition, following seven weeks of exposure to PSNPs, the treated fish group displayed dysregulation of circadian rhythm locomotion activity, which may be associated with melatonin, a crucial hormone regulating the circadian rhythm. This physiological hormone often affects sleep disorders, and it is presently the main treatment for sleep-related pathologies [72]. All of these results proved that PSNP treatments reduced the activity of a number of significant neurotransmitters at the maximum PSNP concentration, which may result in cholinergic neurotransmission insufficiency. These dysregulations increase the likelihood that zebrafish exposure to PSNPs may have a negative impact on neurotransmission. To complete the gene expression analysis, the methylation of these hormones’ genes could be helpful to uncover the molecular mechanism behind the modulation. Mapping hormonal levels among humans to correlate the to NPs exposure is a challenging task. Determining the role of NPs alone could be nearly impossible. Despite this, studies comparing these hormones levels in populations more exposed to NPs to those in less exposed ones could set a starting point. Dosing mRNA levels could also unveil the presence of an early hormonal alteration or a systemic signaling being altered. It is a proven method to use blood mRNA as a biomarker of psychological pathologies [73]. For instance, increased expression of glucocorticoids (such as cortisol) [74], genes enriched for functions of innate-immune response, and interleukins (such as IL-6) [75], mRNA expression of X chromosome gene transcripts [76], and specific miRNA such as microRNA-19b (that was proven to be highly expressed in women exposed to traumas) [77] could all be monitored for the above-discussed hormones (biochemically and genetically). Testing all of these biomarkers could generate a profile of more exposed persons to be correlated with altered behaviors. This aim is challenging and full of bias considering the large number of pollutants that humans are immersed in. To reinforce this assumption, in another study, *zebrafish* larvae were exposed to 25 nm PSNPs (20mg/L from 72 to 120 hpf after hatching) [78]. The results showed that PSNP exposure led to increased release of cortisol-inducing *g6pca* and *pck1* gene expression, altering glucose metabolism. Adults showed hyperactivity when exposed to stress like sudden darkness. The study proved further the connection between PSNP metabolic alterations and hormonal signaling, with consequences on behavior. Despite being microplastics, in another study, PSMP treatment (2 µm) in mice modified the expression of genes and synaptic proteins that are dependent on neuronal activity [79]. It also raised neuroinflammation in the hippocampus, which in turn caused behavioral alterations via the vagus nerve-dependent route. More studies are needed to unveil in detail the exact molecular mechanisms of NP-induced behavioral alterations to define biomarkers of exposure.

### 2.4. NPs and Thyroid Function

**Figure 3:**
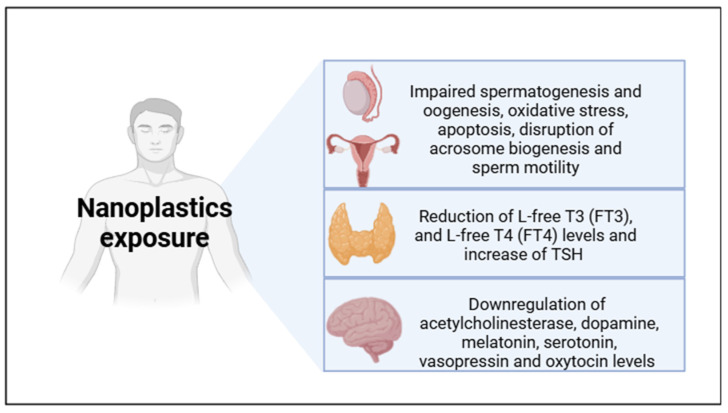
Endocrine effects following NP exposure. Picture designed with Biorender.

In *zebrafish*, no effects induced by NPs alone on thyroid function have been observed. NPs have been proven to exacerbate the effects of other endocrine disruptors such as microcystin-LR (cyanotoxin known for its effects on thyroid) [80] and tris (1,3 dichloro-2-propyl) phosphate (TDCIPP), a thyrotoxic molecule [81]. Conversely, in exposed mice, PSNPs (1, 3, 6, and 10 mg/kg day) were demonstrated to induce a significant reduction in L-free T3 (FT3) and L-free T4 (FT4) levels and an increase in TSH [82]. Considering the relation between thyroid function and the hypothalamus, PSNP effects may disrupt this tissue both directly and indirectly based on the previously discussed evidence on behavior [83]. Figure 3 summarizes the main adverse effects on endocrine functions following NP exposure discussed in this perspective.

## 3. Conclusions

Several papers have proven the presence of bioaccumulated nanoplastics in human tissues [84]. Both in vitro and in vivo studies have correlated it with inflammation and oxidative stress as main drivers of secondary outcomes [85]. In particular, the hypothesis of predisposing individuals to cancer and exploiting genetic predispositions seems to be more and more realistic [86]. Indeed, excessive reactive species and consequent inflammation can alter cellular homeostasis and induce indirectly oxidation of DNA and altered cellular proliferation favoring cancer, as we characterized in our lab on fibroblasts and intestinal cells in vitro [87]. Few studies are focused on the direct effects of nanoplastics on biological targets such as receptors and the consequent endocrine function. The majority describe the effects of plastic leachates but not the role of the particles alone [88]. From the above-mentioned studies, it appears that their specific role should be revalued. In fact, there is a possibility for NPs to module endocrine functions like hormone levels even without being bound to other molecules. Variations in levels of sex and thyroidal hormones and neuroendocrine signals have been observed in different animal models, suggesting a real hazard in humans. To translate the results to humans, more studies are needed that focus on NPs without other pollutants. Bioinformatics could be helpful in predicting specific targets to be investigated. Numerous nanomaterials have been modeled in silico to visualize their physical interaction with endocrine receptors [89,90]. The same approach could be used with NPs as a starting point to be experimented with in vitro or in vivo. The interaction with endocrine receptors may induce an alteration of gene expression depending on the receptor. Finding genetic and molecular biomarkers could help in identifying specific patterns that could define NP exposure or at least help in understanding the adverse effects and eventual palliative treatments. All the studies described focused on biochemical levels. Only a few dig into gene expression, finding, as expected, the overexpression of genes involved in oxidative stress. Therefore, a more detailed approach to oxidative stress to unveil the exact mechanisms that specific polymers and sizes induce may help define specific genes and DNA modifications following NP exposure. Even more challenging is the need to determine epigenetic markers. Studies to analyze whether genes regulated by hormone receptors or related to any endocrine function undergo epigenetic alterations have not been published yet, to our knowledge. Therefore, it could be interesting to visualize the presence of eventual patterns in DNA methylation or histone phosphorylation, as the main epigenetic markers, following NP exposure. Transgenerational effects have been detected and discussed above. Consequently, cells should be able to memorize the presence of NPs and transfer it through generations. Only one paper observed hypo- and hypermethylation of genes involved in diabetes, autoimmune diseases, and cancer [91]. In-depth analysis to unravel whether these “prints” are inheritable and connected to endocrine-related genes could complete the view. Indeed, humans and living organisms in general are exposed continuously to NPs. As discussed in the introduction, NP residues have been found in foods and water in addition to the environmental NPs. Therefore, different exposure routes and timing need to be investigated. Relevance should be given to chronic exposure studies considering the continuous level of exposure that humans are subjected to. To achieve this goal, both in vitro and in vivo models may be useful. For in vitro models, the use of high-resolution microscopy and live imaging together with fluorescent NPs could help in following the toxic effects in real time and also for prolonged periods of time. These technologies may allow the detection of anomalies in sub-cellular structures and changes in macromolecules location and organization [92]. In vivo models may be used to validate in vitro results and to investigate the transgenerational and multigenerational NP-induced effects. The possibility of staring from a parental line and following the offspring may help in unraveling the epigenetic contribution to this topic. In fact, today, only one study has reported epigenetic alterations related to endocrine functions. This is a fundamental aspect to clarify the inheritable toxic effects induced by NPs. The diffusion of omics technologies could help the job. Single cell transcriptomic analysis has already been proven to highlight interesting alterations. In *O. niloticus* gills, 12 cell types have been identified as possible biomarkers of plastics exposure [93]. A similar approach in zebrafish proved that the liver responds, activating stress response pathways with a sex specificity, as summarized in Table 2 [94].

## Figures and Tables

**Table 1:** Endocrine alterations caused by various nanomaterials.

	Tissues	Types of Nanoparticles	Size (nm)	Concentrations	Effects	Ref.
In vitro	Male reproductive system	TiO_2_ Au Eu_2_O_3_	25–70 2.5 9.3–15.4	0–1000 μg/mL 0.5–1.0 × 10^15^ particles/mL 2.5 mg/mL	Reduced spermatogenesis, biosynthesis and catabolic pathways of testosterone, DNA damage in sperm, loss of spermatozoa motility	[41,42,43]
	Female reproductive system	TiO_2_ SiCNWs	50–60 25 80	0–100 μg/mL 12.5–50 μg/mL 0.5–10 μg/mL	Cytotoxicity, reduced viability, increased genotoxicity, inhibition of oocyte maturation and follicle development	[44,45,46]
	Thyroid	Ag, Zn, QDs	2–15	0.1, 5–10 nM	Reduced expression of TH-induced receptor β (TRβ) and TH-repressed Rana larval keratin type I (RLKI)	[47]
In vivo	Male reproductive system	TiO_2_ mPEG@Au	20 33 28.2	0–100 μg/mL 0–1000 mg/kg 45–225 mg/kg	Cytotoxicity, testicular apoptosis, sperm abnormalities	[48,49,50]
	Female reproductive system	TiO_2_	240–280 208–330	0.1, 1 mg/L 10 mg/Kg	Reduced oocyte and follicular maturation, increase of estradiol levels	[51,52]
	Thyroid	Cr	40–70	150, 300, 450 μg/Kg	No effects in THSA, FT3 and FT4 serum levels	[53]

TiO_2_, titanium dioxide; Au, gold; Eu_2_O_3_, europium(III) oxide; SiCNWs, silica carbon nanowalls; Ag, silver; Zn, zinc; QDs, quantum dots; mPEG@Ag, poly (ethylene glycol) coating silver nanoparticles; Cr, chromium.

**Table 2:** Gene and epigenetic dysregulation following NP exposure.

Organisms	Cells and Tissues	Genes	Effects	Ref.
Copepods	Whole body	*Hsp70*, *CuZn SOD*, *CALM3*, *CIDEc* and *p53*	All hypermethylated	[63]
Zebrafish	Oocytes	*sod*, *gpx*, nrf2, inos, *ucp2*, *atp6 nfkβ*, *tnfα*, *il-10*, *ikβ*, *gdf9*, *bmp15*, *gadd45*, *rad51*, *p53* and *bcl2*	All overexpressed	[57]
	Whole body (larvae)	*g6pca* and *pck1*	Both overexpressed	[78]
Rats	Spermatozoa	*Gba2*, *Pick1*, *Gopc*, *Hrb*, *Zpbp1*, *Spaca1* and *Dpy19l2*	*Gopc* and *Dpy19l2* downregulated	[58]
Human	Breast	*TMBIM6*, *AP2M1*, *PTP4A2* and *FTH1*	All overexpressed	[65]
